# Are the new mobile wireless EEG headsets reliable for the evaluation of musical pleasure?

**DOI:** 10.1371/journal.pone.0244820

**Published:** 2020-12-31

**Authors:** Thibault Chabin, Damien Gabriel, Emmanuel Haffen, Thierry Moulin, Lionel Pazart

**Affiliations:** 1 Laboratoire de Neurosciences Intégratives et Cliniques, EA 481, Université Bourgogne Franche-Comté, Besançon, France; 2 INSERM CIC 1431, Centre d’Investigation Clinique de Besançon, Centre Hospitalier Universitaire de Besançon, Besançon, France; 3 Plateforme de Neuroimagerie Fonctionnelle et Neurostimulation–Neuraxess, Centre Hospitalier Universitaire de Besançon, Université Bourgogne Franche-Comté, Besançon, France; European University of Rome, ITALY

## Abstract

Since the beginning of the 20^th^ century, electroencephalography (EEG) has been used in a wide variety of applications, both for medical needs and for the study of various cerebral processes. With the rapid development of the technique, more and more precise and advanced tools have emerged for research purposes. However, the main constraints of these devices have often been the high price and, for some devices the low transportability and the long set-up time. Nevertheless, a broad range of wireless EEG devices have emerged on the market without these constraints, but with a lower signal quality. The development of EEG recording on multiple participants simultaneously, and new technological solutions provides further possibilities to understand the cerebral emotional dynamics of a group. A great number of studies have compared and tested many mobile devices, but have provided contradictory results. It is therefore important to test the reliability of specific wireless devices in a specific research context before developing a large-scale study. The aim of this study was to assess the reliability of two wireless devices (g.tech Nautilus SAHARA electrodes and Emotiv™ Epoc +) for the detection of musical emotions, in comparison with a gold standard EEG device. Sixteen participants reported feeling emotional pleasure (from low pleasure up to musical chills) when listening to their favorite chill-inducing musical excerpts. In terms of emotion detection, our results show statistically significant concordance between Epoc + and the gold standard device in the left prefrontal and left temporal areas in the alpha frequency band. We validated the use of the Emotiv™ Epoc + for research into musical emotion. We did not find any significant concordance between g.tech and the gold standard. This suggests that Emotiv Epoc is more appropriate for musical emotion investigations in natural settings.

## 1. Introduction

Electroencephalography (EEG), which allows the recording of the brain’s electrical activity through the scalp, was developed by Hans Berger in 1929, and has since been a source of great interest for the scientific community. Initially used for neurological or psychiatric diagnosis and in neurosurgery protocols, it undergone rapid developments and is now also used to study specific cerebral mechanisms and how specific areas are related to cognitive functions [1,2]. With the advantage of being transportable, the EEG, which is a direct non-invasive method for cerebral investigations, is one of the most affordable neurophysiological techniques. Moreover, the EEG confers a high temporal resolution with an accuracy to the millisecond that other neuroimaging techniques cannot achieve, and thus provides an interesting alternative to “heavy” techniques such as magnetic resonance imaging or magnetoencephalography [3]. Furthermore, the development of advanced EEG devices has contributed more and more robust tools for studying the relationship between Evoked Response Potentials (ERP) or oscillatory rhythms and cognitive processing. For example, tools such as source reconstruction help to identify the cortical areas involved in specific cognitive processing, but require EEG systems and EEG caps equipped with a large number of electrodes covering the whole scalp. The setup involves the application of gel in order to maintain the best impedance quality. Consequently, these still have a low transportability [4], are expensive, and take a long time to set up.

In the past decade, a few mobile EEG headsets have been introduced onto the EEG market (for a broad review see [5,6]). Advantages of these new technologies include quick and easy positioning due to dry or saline electrodes [7], wireless Bluetooth or Wi-Fi data transmission, more mobility [8] and, last but not least, affordable prices. The range of products extends from the most simple consumer headsets equipped with only a few electrodes, such as Emotiv™ or Neurosky™ products [9–12], to reliable devices for research works, such as g.tech, Cognionics, or Neuro-electrics products. The former products are less expensive, ranging from $ 300 to $ 900, and are intended for home use and Brain Computer Interface (BCI) applications [10,13,14]. In contrast, the latter products are more robust, provide a better signal/noise ratio, and are usually used in research protocols or for medical applications. However, they remain very expensive, costing up to $ 40,000 [15].

One potential application of the mobile EEG tools, which has already been developed by a number of teams, is in hyperscanning paradigms, in which the cerebral activity of multiple participants is recorded simultaneously in various contexts, both in laboratory or ecological/natural settings [16–30]. In light of this, compared to advanced EEG research tools, the most affordable devices provide new possibilities to work with larger samples of participants. As the field of social neuroscience moves toward ‘real-world neuroscience’ [31,32], these developments open up a new area of research, with the implementation of paradigms in ecological or natural conditions in group settings [19,20]. This is all the more important for research into musical emotion, in which stimulations and situations need to be as ecological as possible and in which the social aspects of concert settings could greatly influence the emotional experience [33]. To study how musical emotions are shared between people with objective measures of cerebral activity, in natural or ecological conditions, would advance the understanding of the mechanisms underlying musical emotional communication [34–37]. Several studies have already investigated neural synchronization during social engagement tasks [20] or during interpersonal coordination of musicians [22,24,27]. However, the cerebral synchronization of multiple people in the context of music emotion has never been investigated, and its study should lead to progress in understanding group emotional dynamics. Recording the cerebral activity of several people simultaneously in a natural setting while they are listening to emotional music should partially cover this gap [34], despite being a very challenging prospect. A previous study assessed the feasibility of such a paradigm in a concert [34] and suggested a good adherence of the majority of public participants and musicians. Now, technical constraints and devices should be tested to ensure that musical emotion can be measured by wireless mobile devices with dry or gel-free electrodes.

Our main interest is the physical differences between the hardware, which include different electrode types and different data transmissions. Wireless communication is more subject to parasites and global environmental noise than shielded cable communication [38]. Most mobile wireless devices are equipped with dry electrodes (comb-like and multi-pin electrodes [3,39] as for g.tech nautilus SAHARA electrodes) or saline-based wet-contact resistive electrodes as for Epoc + [40]. Compared to classic Ag/AgCl electrodes—which are saturated with gel or saline electrolyte solution for better electrical conduction, thus keeping the best impedance through the experiment—a major concern in using dry electrodes is obtaining the best signal for research purposes [3]. Since wireless wet-based or dry electrodes devices have not been widely used for investigations into musical emotion, it appears crucial to test the targeted devices in such a paradigm before implementing complex natural setting experiments in large groups. With EEG, musical emotional pleasure is measured mostly on frontal electrodes in the alpha and theta frequency bands. Frontal theta oscillations are involved in musical appreciation [41] and while listening to pleasant music [42]. The overall frontal alpha activity is reported to be a reliable indicator of musical emotions [13,43,44]. In light of these results, the relevance of specific theta and alpha oscillatory rhythms recorded with wireless devices that are equipped with a low number of dry electrodes in the measurement of musical emotion/pleasure is still an open question.

The aim of this study was to compare wireless mobile EEG devices equipped with dry or saline-based wet-contact resistive electrodes with a gold standard EEG device equipped with Ag/AgCl electrodes and a wire transmission, for the detection of musical pleasure while listening to chill-producing musical extracts. Since the studies of musical emotion and musical pleasure found in literature were performed in very varied paradigms and types of analysis, it is difficult to formulate a clear hypothesis about the expected oscillatory activity. This is a major concern, especially since we know that the context, the experimenter instructions, the study design and the reference electrode influence the signal and results. Consequently, the principal judgment parameter for the comparison was to find similar oscillatory rhythms and similar activities for corresponding electrodes/regions of interest for each device with the gold standard EEG.

In light of the results of our recent study on musical chills with EEG [35] and other research on musical pleasure [42,45–47], our main hypothesis was formulated for frontal activity. We expected that we would observe a gradual increase of theta power spectral density (PSD) values with the increase of emotional pleasure in the frontal/prefrontal areas with both devices. Moreover, we expected a gradual decrease of theta PSD values on temporal and central sites as well as a gradual increase of the beta/alpha ratio, associated with high emotional arousal [13,48], during the increase of emotional pleasure.

## 2. Materials and methods

### 2.1 Procedure

#### 2.1.1 Ethics

The study was approved by an independent ethics committee (CPP Ouest V–Rennes; n° 2018-A01653-52) and follows recommendations from the French Jardé Law (Article R1121-1 1 of the French Public Health Code, amended by decree 127 n° 2017–884 of May 2017) concerning non-invasive protocols involving healthy humans. All participants received the study information in fully, both oral and written, and signed a written informed consent form before their inclusion. The participants each received € 25 for their participation. The study is listed on ClinicalTrials.gov under the identifier: NCT03753230.

#### 2.1.2 Data availability statement

The datasets presented in this study can be found in online repositories. The names of the repository/repositories and accession number(s) can be found below: CHABIN, Thibault; Gabriel, Damien; Haffen, Emmanuel; Moulin, Thierry; Pazart, Lionel (2020): Are the new mobile wireless EEG headsets reliable for the evaluation of musical pleasure?. figshare. Dataset. https://doi.org/10.6084/m9.figshare.12936800.v1

#### 2.1.3 Paradigm

For inclusion in the study, participants needed to be sensitive to musical reward. In that respect, they were expected to regularly experience chills while listening to music and to score higher than 65 on the French version of the Barcelona Music Reward Questionnaire (BMRQ; Mas-Herrero et al., 2013; Saliba et al., 2016 [49,50], score above 65: classified as Hedonic/Hyperhedonic to musical reward according to Mas-Herrero et al., 2014) [51]. They were also asked to provide at least seven musical extracts that frequently produce high pleasure up to musical chills. No specific criteria were defined for musical extract selection, all musical styles were accepted.

**Fig 1:**
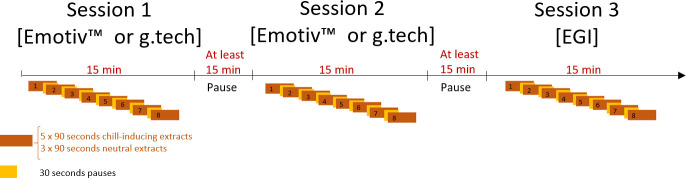
Flow chart of the experiment.

Of the extracts they provided, five were selected and the participants were asked to indicate in advance the precise moment that produced the most intense peak pleasure and triggered musical chills. Their extracts were cut to 90 seconds starting 60 seconds before the indicated peak pleasure (Fig 1), normalized to 0 dB and faded in/out. During the listening session, participants were asked to report their subjective felt pleasure according to four levels: (1) neutral, (2) low pleasure, (3) high pleasure and (4) chills. They were asked to maintain the pressure on the relevant button and to indicate each new chill even if they were already pushing the chill button. They were asked to report only chills associated with intense pleasure and physical sensations such as goose bumps, hairs standing on end, chills, shivers or tingling sensations down the spine, and so on. Participants performed three successive listening sessions with the Emotiv™ Epoc device, g.tech Nautilus SAHARA, and the EGI system in a random order (see flow chart). In each listening session, they listened to the same eight extracts including five extracts they provided and three neutral extracts selected by experimenters. The additional neutral extracts had been previously selected by an independent sample of participants (for more details on the method see [29]). Pauses of 30 seconds were inserted between the extracts.

### 2.2 Participants

Among the 90 participants that contacted us, 58 did not fulfill the inclusion criteria (to score higher than 65 for the BRMQ, to be exclusively right-handed, to provide at least 7 chill-inducing extracts, to send back questionnaires after the initial request for information). For some participants we could not program an appointment and several were not re-contacted because the inclusion period had passed. Finally, 23 subjects passed a medical examination to confirm they had normal hearing (with audiogram tests) and an absence of neurological or psychiatric disorders. These 23 participants were recruited for the study.

However, for some participants, not enough chills were reported for one of the three listening sessions. Consequently, the EEG results presented here concern the comparison of data acquired for participants that reported enough chills, giving enough acceptable epochs of 1 second in both sessions (Gold standard and Emotiv™ or Gold standard and g.tec, see Table 1).

**Table 1:** Demographic data.

Participant	Age/Sex (Male/Female)	Global score BMRQ	Musical practice (years)	n chills (EGI)	n chills (Epoc +)	n chills (g.tech)	Concordance Analysis
S01	37/F	69	12	15	13	25	3
S02	28/M	88	15	19	18	17	3
S03	22/F	88	15	16	4	8	2
S04	21/F	85	15	19	15	18	2
S05	31/F	91	22	48	29	12	3
S06	59/M	78	0	50	41	32	3
S07	36/F	83	29	8	7	8	3
S08	37/F	83	0	24	6	25	1
S09	30/F	76	15	11	7	8	3
S10	24/M	84	14	17	25	2	1
S11	38/M	81	10	6	24	13	1
S12	43/M	72	0	7	2	6	2
S13	18/F	79	0	9	8	21	3
S14	61/F	84	0	4	4	6	3
S15	73/F	73	0	13	14	12	3
S16	72/M	91	55	14	21	11	3

(For the column “Concordance”; [1] indicates that the participant has been included in the analysis of Epoc + vs EGI, [2] analysis of g.tech vs EGI, [3] analysis of both Epoc + and g.tech vs EGI.).

Finally, 16 subjects (6 men/10 women) with a mean age of 39.3 years (SD = 17.7, range = 18–73) were included in the analysis. Ten were amateur musicians with a mean of 12.6 years of practice (SD = 14.4; range = 10–55). Thirteen participants were included in the concordance analysis between the Emotiv™ Epoc + headset and the gold standard, and 13 participants were included in the concordance analysis between the g.tech and the gold standard based on the number of chills free from artefact epochs we were able to keep in both analyses (see Table 1).

### 2.3 EEG recordings

#### 2.3.1 Gold standard EEG

High-Density EEG recordings were performed using Netstation tools (V5.4) with Net Amp 300 high- impedance amplifiers from Electrical Geodesic **(EGI)** and a 256-channel Sensor Net composed of saline based Ag/AgCl electrodes. The frequency range was fixed at 1000 Hz and a high pass fixed at 1 Hz was applied during the recording session. During the experiment, the impedance was kept under 50 KΩ. All channels were referenced to vertex (Cz).

#### 2.3.2 Mobile EEG headset

**Fig 2:**
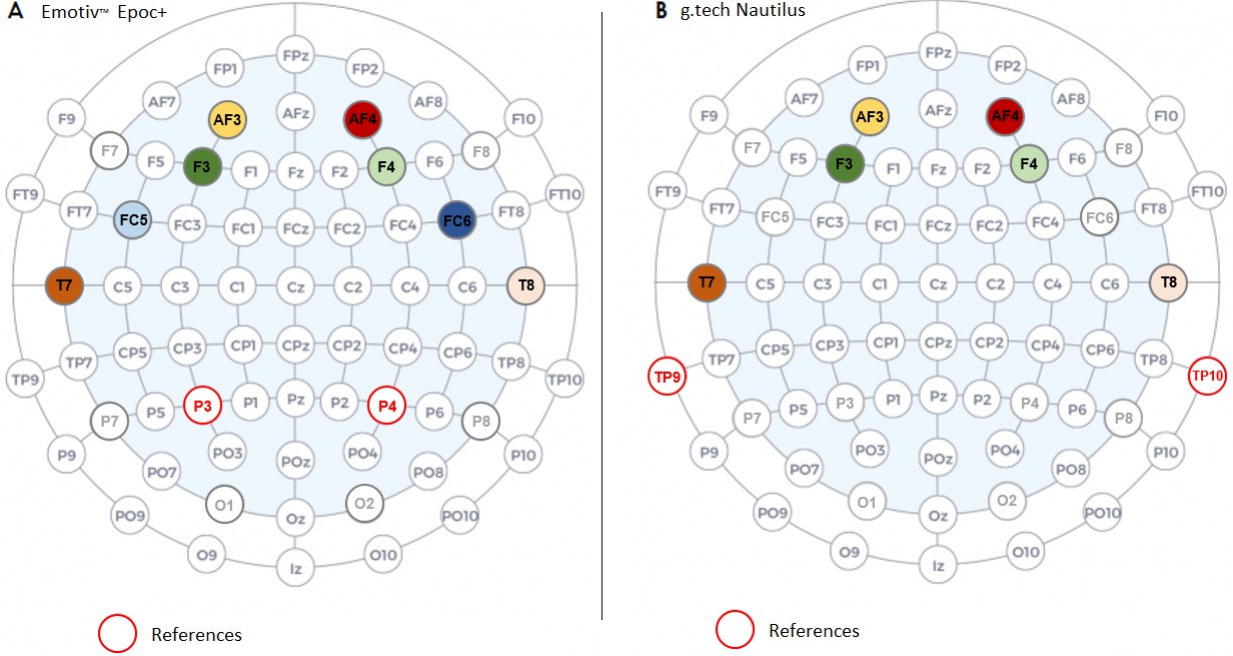
[A] Positioning of the Epoc + channels included in the analysis over the scalp according to the 10–20 system, [B] Positioning of the g.tech electrodes included in the analysis over the scalp according to 10–20 system.

***Emotiv***™ ***Epoc +*.** EEG data were recorded using the wireless EEG headset Emotiv™ Epoc + (version 2018 and 2019). This headset is composed of 14 usable saline electrodes organized according to the 10–20 system (F3, F4, AF3, AF4, F7, F8, FC5, FC6, O1, O2, T7, T8, P7, P8) and 2 references on parietal sites (P3 and P4) (Fig 2). The recordings were performed with the sampling rate fixed at 128 Hz. For more details, see the technical specifications at the following address: https://www.emotiv.com/product/emotiv-epoc-14-channel-mobile-eeg/#tab-description. During data acquisition, no traditional values are given for impedance measures, instead the contact quality is given (0 = no contact, 1 = low contact quality, 2 = medium contact quality, 3 = good contact quality, 4 = excellent contact quality). All electrodes were kept on 4 during the experiment.

***g*.*tech Nautilus SAHARA Electrodes*.** EEG data were recorded using a second wireless EEG system, i.e. the g.tech Nautilus with eight SAHARA dry pin electrodes (AF3, AF4, F3, F4, F7, F8, T7, T8) referenced with adhesive electrodes on the mastoids (TP9, TP10) (Fig 2). The recordings were performed using OpenVibe (V2.2.0) with the sampling rate fixed at 250 Hz. For more details, see the technical specifications at the following address: https://www.gtec.at/product/gnautilus-research/. Impedance was checked at the beginning of the recording using g.Need Access API. Due to the pin electrodes, which do not require gel or saline solution, the impedance cannot be improved.

### 2.3.3 Analysis

We used Cartool Software (version 3.7) to pre-process all the EEG data. For the concordance analysis with EmotivEpoc +, the EGI data were resampled to 128Hz and re-referenced to P3 & P4. For the concordance analysis with g.tech, EGI data were resampled to 250Hz and re-referenced with TP9 & TP10. We applied a 30-Hz low-pass filter and 1-Hz high-pass filter to all the data (butterworth, 2^nd^ order), in addition to a notch filter at 50 Hz. We then divided the data into 1-second epochs according to three levels of emotion: (1) Low pleasure, (2) High pleasure, (3) Chills. Epochs from Neutral and Low pleasure were grouped together in the condition “Low Pleasure” since participants did not distinguish between these two levels of emotion. All epochs were visually inspected and epochs not free from artefacts (motor, visual or muscular artefacts) were rejected. For the 13 subjects that were included in the concordance analysis between Emotiv and EGI, Wilcoxon tests revealed no significant difference in the number of epochs rejected (EGI = 29.5%, SD = 10.4; Emotiv = 29.4%, SD = 11.8; W(13) = 44, p = 0.94). For the 13 subjects that were included in the concordance analysis between g.tech and EGI, Wilcoxon tests also revealed no significant difference in the number of epochs rejected(g.tech = 23.1%, SD = 10.2; EGI = 24.4%, SD = 11.4; W(13) = 42, p = 0.83). A Fast Fourrier Transform was performed using Matlab (version 2019a), on each epoch of each condition according to three frequency bands; Theta (4–8 Hz), Alpha (8-12Hz), Beta (12–20 Hz). We used the Welch method (Hanning window, 50% overlap) based on FFT magnitude squared to estimate the Power Spectral Density (PSD).

**Fig 3:**
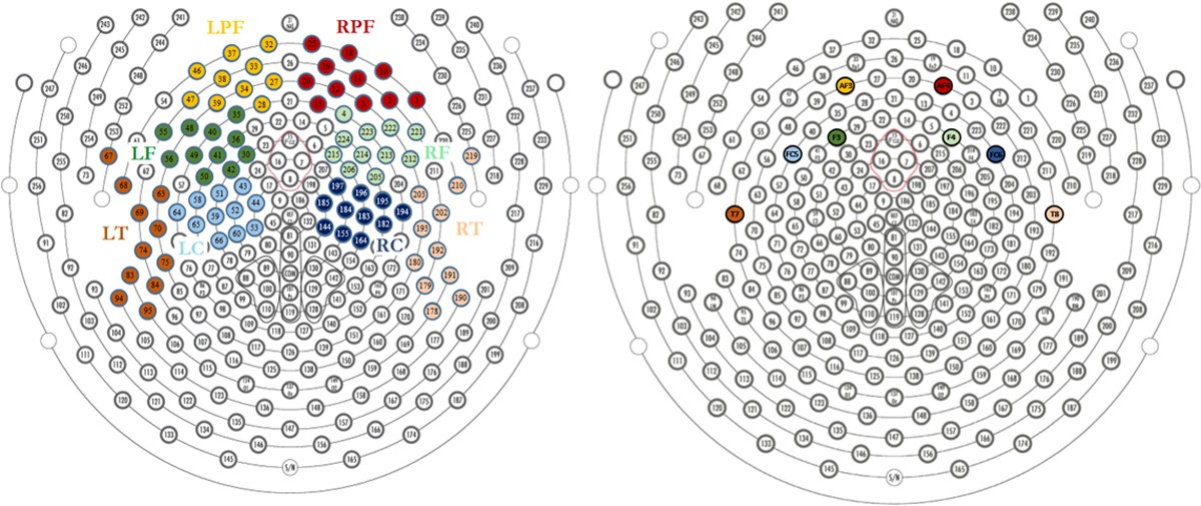
[A] Large Regions of interest for EEG analysis, Left Prefrontal (LPF), Right Prefrontal (RPF), Left Frontal (LF), Right Frontal (RF), Left Temporal (LT), Right Temporal (RT), Left Central (LC), Right Central (RC). [B] Corresponding electrodes for Epoc + and g.tech devices (AF3, AF4, F3, F4, FC5, FC6, F7, F8, T7, T8).

Large regions of interest (ROI) were made, each including 10 electrodes (for EGI); Left Prefrontal ROI (LPF ROI) matching with AF3, Right Prefrontal ROI (RPF ROI) matching with AF4, Left Frontal ROI (LF ROI) matching with F3, Right Frontal ROI (RF ROI) matching with F4, Left Central ROI (LC ROI) matching with FC5, Right Central ROI (RC ROI) matching with FC6, Left Temporal ROI (LT ROI) matching with T7, and Right Temporal ROI (RT ROI) matching with T8. (See Fig 3).

For the data from both devices, we calculated the beta/alpha ratio for the prefrontal and frontal area/electrodes that give a direct measure of Arousal that represent the strength of the emotion [52]. We applied this calculation to the AF3, AF4, F3, and F4 electrodes for the Epoc + and g.tech devices (***Arousal 1***), and to the prefrontal and frontal ROIs for the EGI device (***Arousal 2****)*.

$$Arousal\;1=\frac{\left(\beta \;\text{AF}3+\beta \;\text{AF}4+\beta \;\text{F}3+\beta \;\text{F}4\right)}{\left(\alpha \;\text{AF}3+\alpha \;\text{AF}4+\alpha \;\text{F}3+\alpha \;\text{F}4\right)}$$

$$Arousal\;2=\frac{\left(\beta \;\text{ROI}\;\text{LF}+\beta \;\text{ROI}\;\text{RF}+\beta \;\text{ROI}\;\text{RPF}+\beta \;\text{ROI}\;\text{LPF}\right)}{\left(\alpha \;\text{ROI}\;\text{LF}+\alpha \;\text{ROI}\;\text{RF}+\alpha \;\text{ROI}\;\text{RPF}+\alpha \;\text{ROI}\;\text{LPF}\right)}$$

Finally, an Alpha to total Power ratio (***Alpha/Total PSD*** with ***Total PSD*** the sum of delta, theta, alpha, beta PSD over F3 and F4) was calculated for chills epochs for EGI and g.tech at 250Hz and EGI. For EGI the corresponding ROIs were used.

$$Alpha/Total\;PSD=\frac{\left(\alpha \;\text{F}3+\alpha \;\text{F}4\right)}{\left(\delta \;\text{F}3+\delta \;\text{F}4+\theta \;\text{F}3+\theta \;\text{F}4+\alpha \;\text{F}3+\alpha \;\text{F}4+\beta \;\text{F}4+\beta \;\text{F}4\right)}$$

#### 2.3.4 Statistics

We performed all statistical analyses using R Studio software (version 3.5.2; 2018-12-20). For behavioral data, we compared the number of reported chills in the listening sessions using Friedman tests. To check the potential bias of subject demographic characteristics on reports of chills, we applied Spearman's Rank-Order Correlation to the age; number of years of musical practice, BMRQ sum of items, and the number of chills reported in each session. We applied Friedman tests to compare the Power Spectral Density values of the three levels of emotion (Low pleasure, High pleasure, Chills), for Arousal calculations and for alpha and Theta frequency bands on all ROI. We applied a paired samples Wilcoxon test to compare each condition for each ROI (EGI) and each electrode (Epoc + and g.tech). We applied a correction of the p value according to the multiplicity of tests (by frequency band) (Emotiv™ vs EGI; p value correction to 0.05/8 = 0.0063, g.tech vs EGI; p value correction to 0.05/6 = 0.0083). Alpha to Total PSD ratios were compared for g.tech versus EGI and Emotiv™ vs EGI with Wilcoxon tests.

## 3. Results

### 3.1 Behavioral data

There were no differences in the number of reported chills between the listening sessions (Fr(2.16) = 3.77; p = 0.15). Spearman correlations did not show any significant relationship between demographic characteristics and the number of reported chills (*n chills Emotiv session vs age*: p = 0.93, r_s_ = 0.02; *n chills EGI session vs age*: p = 0.34, r_s_ = -0.25, *n chills g*.*tech session vs age*: p = 0.86, r_s_ = -0.04; *n chills Emotiv session vs sum items BMRQ*: p = 0.31, r_s_ = 0.26; *n chills EGI session vs sum items BMRQ*: p = 0.18, r_s_ = 0.34; *n chills g*.*tech session vs sum items BMRQ*: p = 0.50, r_s_ = -0.17; *n chills Emotiv session vs n years of musical practice*: p = 0.35, r_s_ = 0.24; *n chills EGI session vs n years of musical practice*: p = 0.50, r_s_ = 0.18; *n chills g*.*tech session vs n years of musical practice*: p = 0.38, r_s_ = -0.23).

### 3.2 Concordance analysis Epoc + vs EGI

#### 3.2.1 Epoc + Results

**Fig 4:**
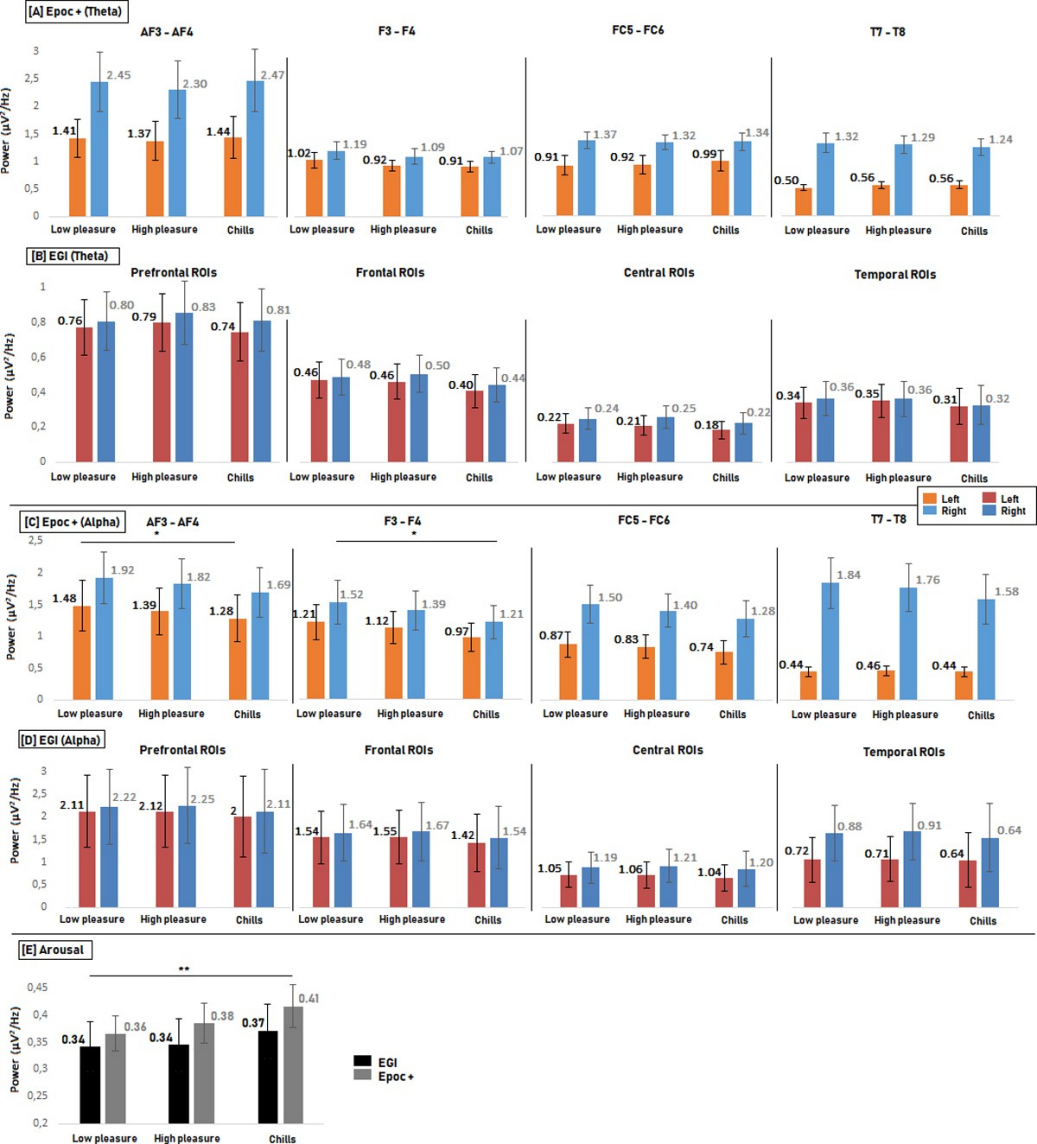
Comparison of PSD values for each condition (Low pleasure vs High pleasure vs Chills) for each electrode of the Epoc + headset [A] and [C] and matching ROI for EGI [B] and [D] in the theta and alpha frequency bands. (Error bars represent the standard error, * < 0.0063). Comparison of PSD values for each condition for Arousal calculation [E] (Arousal 1; prefrontal and frontal electrode (Epoc +) and Arousal 2; prefrontal and frontal ROIs (EGI). Error bars represent the standard error,* < 0.025, **<0.005.).

For the theta frequency band, the comparison of each level of emotional pleasure using Friedman tests did not show significant differences for any electrodes (all p values > 0.19) and we identified no meaningful trends (see Fig 4[A]).

For the alpha frequency band, the comparison of each level of emotional pleasure showed significant effects for electrodes AF3 (Fr(2,13) = 6; p<0.05), AF4 (Fr(2,13) = 6.61; p<0.05), F4 (Fr(2,13) = 9.84; p<0.01), T7 (Fr(2,13) = 6; p<0.05), and FC6 (Fr(2,13) = 9.84; p<0.01). In each case, the higher the emotional pleasure, the lower the PSD value (see Fig 4[C]). For electrodes F3, FC5, and T8, no significant differences between conditions were found (p>0.06), but for each case, the lower the PSD value, the higher the emotional pleasure. The post-hoc test with correction of the p value (p = 0.0063) showed a significant effect between the “Low pleasure” and “Chills” conditions for F4 and AF3 (p = 0.0018 and p = 0.0057 respectively) in the alpha frequency band. Given the exploratory nature of this study and the absence of preliminary results for musical pleasure detection with such references (P3 and P4), we explored the post-hoc effect without correction of the p value by frequency band (p = 0.05). Without p value correction, significant differences were found between the “Low pleasure” and “Chills” conditions for electrodes AF3 (p = 0.005), AF4 (p = 0.01) F3 (p = 0.007), F4 (p = 0.0018), FC5 (p = 0.023), and FC6 (p = 0.007). There was also a significant difference between “Low pleasure” and “High pleasure” for the F4 electrode (p = 0.039) and the FC6 electrode (p = 0.039).

For the ***Arousal 1*** calculation, the higher the PSD ratio, le higher the pleasure (Fr(2,13) = 8; p < 0.05), with a significant difference between “Low pleasure” and “Chills” (p = 0.004) (see Fig 4[E]).

All the results are summarized in Table 2.

**Table 2:** Summary of statistics for each EGI ROI and the corresponding electrode for the Epoc +.

Frequency band	Device	ROI LPF AF3	ROI RPF AF4	ROI LF F3	ROI RF F4	ROI LC FC5	ROI RC FC6	ROI LT T7	ROI RT T8
**Alpha**	**EGI** Stat *Friedmann* (p value)	6(0.049)	2 (0.36)	3.2 (0.19)	2.46 (0.29)	4.76 (0.09)	3.84 (0.14)	6(0.049)	3.84 (0.14)
	**Epoc +** Stat *Friedmann* (p value)	6.61(0.036)	6.61(0.036)	5.53 (0.062)	$\begin{array}{c} 9.84\;\left(0.007\right) \end{array}$	4.76 (0.09)	$\begin{array}{c} 9.84\;\left(0.007\right) \end{array}$	6(0.049)	1.07 (0.58)
**Theta**	**EGI** Stat *Friedmann* (p value)	2.92 (0.23)	5.53 (0.062)	2 (0.36)	2.46 (0.29)	3.2 (0.19)	4.7 (0.092)	1.07 (0.58)	5.69 (0.058)
	**Epoc +** Stat *Friedmann* (p value)	0.61 (0.73)	2.46 (0.29)	0.46 (0.79)	3.2 (0.19)	0.46 (0.79)	0.15 (0.92)	2 (0.36)	0.61 (0.73)
**Arousal 1 (Epoc +)** Stat *Friedmann* (p value)		8(0.018)							
**Arousal 2 (EGI)** Stat *Friedmann* (p value)		1.07 (0.58)							

(All results are referenced to P3 & P4 and concern the 13 participants who experienced enough chills with both EGI and Emotiv).

(Green cells indicate consistent effects).

#### 3.2.2 EGI results

For the theta frequency band, the comparison between all levels of emotional pleasure (Low pleasure, High pleasure and Chills) using Friedman test did not show significant differences between conditions for any region of interest (Fig 4[B]), and we identified no meaningful trends.

For the alpha frequency band, the comparison between all conditions (low pleasure, high pleasure and chills) revealed a significant effect for the left prefrontal ROI (Fr(2,13) = 6; p<0.05) and the left temporal ROI (Fr(2,13) = 6; p<0.05). In both cases, the lower the PSD, the higher the emotional pleasure (Fig 4[D]). For the right prefrontal ROI, frontal and central ROIs, and right temporal ROI there were no significant differences between conditions (p>0.1). Nevertheless, we observed the same trend. More precisely, there the lower the PSD values, the higher the emotional pleasure. Post-hoc tests after p-value correction did not show significant effects for the left prefrontal and left temporal ROIs. These post-hoc tests without p-value correction (p = 0.05) showed a significant effect between the “Low pleasure” and “Chills” conditions for the left prefrontal ROI (p < 0.05), and for the left temporal ROI (p < 0.05) in the alpha frequency band.

For the ***Arousal 2*** analysis, there was a non-significant increase in the Beta/Alpha PSD value (Fr(2,13) = 1.07; p = 0.58) (Fig 4[E]).

### 3.3 Concordance Analysis g.tech vs EGI

#### 3.3.1 g.tech results

**Fig 5:**
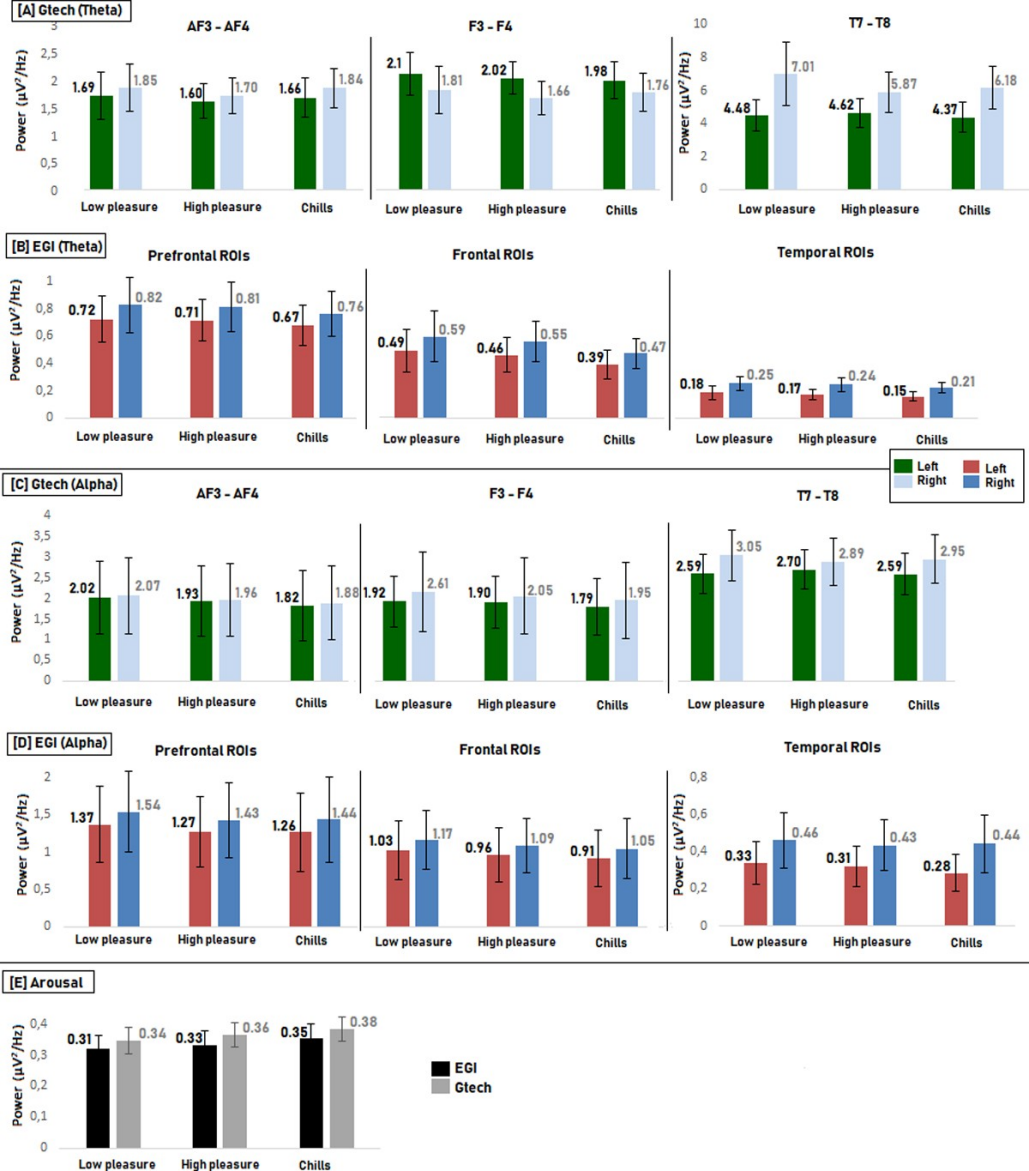
Comparison of PSD values for each condition (Low pleasure vs High pleasure vs Chills) for each electrode of the g.tech headset [A] and [C] and matching ROI for the EGI [B] and [D] in the theta and alpha frequency bands. (Error bars represent the standard error). Comparison of PSD values for each condition for Arousal calculation [E] (Arousal 1; prefrontal and frontal electrode (g.tech) and Arousal 2; prefrontal and frontal ROIs (EGI). Error bars represent the standard error).

For both the alpha and theta frequency bands and for arousal analysis, there were no significant effects when comparing the different levels of emotion using Friedman tests (Fig 5[A] and 5[C]). We identified no specific trends for any electrode for the theta band, but we observed a decrease of the PSD values in the alpha band on frontal and prefrontal electrodes the higher the emotion. For the ***Arousal 1*** calculation, we observed no significant difference between the different emotional levels but the PSD values increased the higher the pleasure (Fig 5[E]).

All the results are summarized in Table 3.

**Table 3:** Summary of statistics for each EGI ROI and the corresponding electrode for the g.tech.

Frequency band	Device	ROI LPF AF3	ROI RPF AF4	ROI LF F3	ROI RF F4	ROI LT T7	ROI RT T8
**Alpha**	**EGI** Stat *Friedmann* (p value)	2 (0.36)	2.46 (0.29)	3.2 (0.19)	3.2 (0.19)	3.7(0.018)	2 (0.36)
	**g.tech** Stat *Friedmann* (p value)	1.38 (0.5)	2 (0.36)	0.61 (0.73)	0.15 (0.92)	1.07 (0.58)	2.92 (0.23)
**Theta**	**EGI** Stat *Friedmann* (p value)	2.46 (0.29)	1.07 (0.58)	$\begin{array}{c} 7.53\;\left(0.023\right) \end{array}$	1.84 (0.39)	2.92 (0.23)	2 (0.36)
	**g.tech** Stat *Friedmann* (p value)	0.61 (0.73)	2.92 (0.23)	2 (0.36)	0.15 (0.92)	2.92 (0.23)	2 (0.36)
**Arousal 1 (g.tech)** Stat *Friedmann* (p value)		1.84 (0.39)					
**Arousal 2 (EGI)** Stat *Friedmann* (p value)		4.3 (0.11)					

(All results are referenced to TP9 & TP10 and concern the 13 participants who experienced enough chills with both g.tech and EGI).

#### 3.3.2 EGI results

For the theta frequency band, we observed a decrease in PSD value the higher the emotion for all ROIs. Friedman tests showed a significant difference over the Left Frontal ROI; the higher the emotion, the lower the PSD values (Fr(2,13) = 7.53; p = 0.023). We identified no significant effects for all other regions of interest. The post-hoc tests with p-value correction (p = 0.0083) did not reveal significant differences between conditions. Nevertheless, without p-value correction there was a significant difference between the “High pleasure” and “Chills” conditions (p = 0.013) (Fig 5[B]).

For the alpha frequency band, a general decrease in PSD values was also observed when the higher emotional pleasure for all ROIs (except the Right Temporal ROI). We observed a significant difference for the Left Temporal ROI; the higher the emotional pleasure, the lower the PSD value (Fr(2,13) = 3.7; p = 0.018). Post-hoc tests with p-value correction did not reveal significant differences. Without p-value correction there was a significant difference between Low Pleasure vs High Pleasure (p = 0.03) and between Low Pleasure vs Chills (p = 0.04) (Fig 5[D]).

For the ***Arousal 2*** calculation, the higher the PSD values, the higher the emotional pleasure, but the Friedman test did not reveal significant differences (Fig 5[E]).

### 3.4 Alpha to total power spectral density

**Fig 6:**
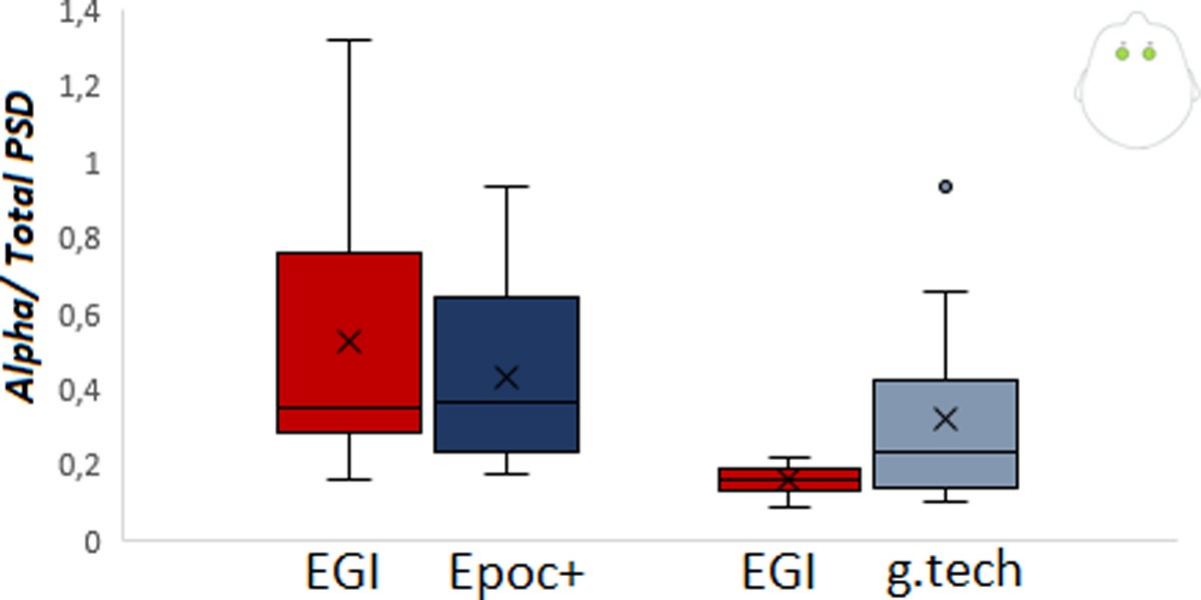
Alpha/Total PSD (Delta, Theta, Alpha, beta power) for chill epochs over frontal sites both the comparisons of Epoc+ vs EGI and g.tech vs EGI.

The alpha to total spectral power ratio for chills epochs over frontal sites was not significantly different for EGI vs Epoc + (W(13) = 59, p = 0.37) or EGI vs g.tech, although the later comparison was close to the significance threshold (W(13) = 73, p = 0.057) (see Fig 6).

## 4. Discussion

The aim of this study was to assess the reliability of the g.tech and Emotiv™ Epoc + wireless EEG headsets equipped with dry or saline-based wet-contact resistive electrodes in comparison with a wire gold standard medical EEG device (EGI Net Amp 300) equipped with Ag/AgCl electrodes. A total of 16 participants listened to their favorite pleasurable musical extracts that regularly produce chills. Results of the concordance analysis showed concordant effects for the EGI and Epoc + devices for the left prefrontal ROI and AF3 electrode and for the left temporal ROI and T7 electrode in the alpha frequency band. Considering the exploratory nature of the study, without p-value correction, post-hoc tests gave significant consistent effects for both devices in the left prefrontal ROI and the AF3 electrode. For the concordance analysis of the g.tech device with EGI, the effects found over the Left Frontal ROI in the theta frequency band and over the Left temporal ROI in the alpha band for EGI devices were not observed for g.tech devices. However, the same trends (decreased PSD value, the higher the emotional pleasure) were observed for both devices, especially in the alpha frequency band for frontal and prefrontal areas. For both concordance analyses, the alpha to total spectral power ratio was not significantly different on frontal sites (even if a result close to the significance threshold was been found for g.tech vs EGI). This result suggests, at least for the Emotiv headset, that the oscillatory rhythms recorded with both devices are not physically different in this condition.

In general terms, the majority of comparisons between the three levels of emotional pleasure for the Epoc + concordance analysis showed the same trends for both devices even in the absence of a statistically significant concordance. This is a predominant observation for the interpretation of results. Moreover, even if we did not calculate the asymmetry indexes that compare the power ratio between the right vs the left hemispheres, our results show that for both devices the activity is higher for the right than for the left hemisphere in the alpha band. The comparison of PSD amplitudes for both levels of emotion is therefore an argument in favor of the validity of the Epoc + system in this paradigm. For the theta frequency band, the absence of significant and meaningful trends for both devices reinforce this validity. We observed no gradual increase and no gradual decrease of activity that would represent meaningful processing. In other terms, with the referencing to P3 and P4 for both devices, there is no theta oscillatory activity that can be attributed to the measure of musical pleasure. For arousal even in the absence of significant effects for EGI devices, both devices recorded an increased beta/alpha ratio on prefrontal and frontal areas, the higher the emotional pleasure. These results suggest that the emotional musical pleasure was measured with both devices. These result are also concordant (for both devices) with physiological data from Rickard [53] and Salimpoor [54], suggesting that an increasing pleasure and the onset of chills produce an activation of the peripheral system with higher skin conductance responses/levels and higher heart rate levels. Our previous research did not demonstrate the relevance of approach withdrawal analysis with alpha asymmetry calculations with the EGI gold-standard setup [35]. Consequently, we chose to analyse only alpha and theta PSD measures. Furthermore, the comparison of relative alpha power on frontal sites for chills epochs reinforced the robustness of the headset comparison. Although we did not find a more significant effect for EGI, in general terms the Epoc + signal was noisier. Compared to EGI, the Epoc + is not protected by a Faraday cage, which can influence the signal as well as the wire transmission. While the number of rejected epochs was did not differ significantly between the devices, we concluded that Epoc+ still appears less performant since there are only 14 electrodes, which drastically reduces the risk of artifacts compared to 256 electrodes. This lower performance is likely to be due to the specific wet-contact resistive electrodes.

For g.tech concordance analysis with EGI, the ratio of PSD values between the left and right hemisphere are the same for both devices (except for frontal areas in theta) and the arousal calculations show the same trends. Despite the general overall decrease of the PSD values the more the emotional pleasure was high for both devices, the effects found for the EGI device were not reproduced. In this case, it is not the quality of the device but the type of electrode that raises questions. Indeed, a study compared the g.tech Nautilus, with its 8-electrode configuration but with the GAMMA version of electrodes (with gel application) to a BioSemi device equipped with 64 channels [4]. They found that there were no significant differences in peak latency and amplitude on the Fz, Cz, and Pz electrodes between devices during a P300 ERP for a go/no go task. In the present study, the specific type of pin electrodes, which have a low contact with the skin, without saline solution or gel-based application seems to need further developments. We hypothesize that the inability to improve impedance when the headset was set up influenced the quality of signal and potentially influenced the results. In this case, without consistent significant effects, the common absence of effect is not a solid base for the validation of this system with SAHARA pin electrodes for the detection of musical pleasure. Indeed g.tech have developed a hybrid gel-based pin electrode that replaces the metal pin electrode without gel.

The results presented here for both devices are quite different compared to results from our first study about the detection of musical pleasure using EEG (in the same paradigm with the EGI device; under review). The major difference is found in the theta frequency band, especially for the prefrontal sites for which we observed an increase in the PSD value the higher the emotional pleasure. The present results do not validate this hypothesis about theta oscillatory activity. Although the theta activity reflects the musical pleasure processing [35,42,45–47], this frequency band is also involved in other cognitive processes such as mental effort [55,56]. Consequently, whether theta is also involved in mental effort here should be investigated further. With an average referencing, theta power activity on prefrontal sites increased the higher the emotional pleasure, whereas with P3/P4 or TP9/TP10 referencing this activity decreased. However, these contradictory results were expected and can be easily explained. For EEG recordings, there is no electrode that records zero potential, and so changing the reference will change “the waveform shapes at each recording electrode” [57]. Statistical comparison of amplitude for different conditions will therefore change if the reference is changed [58]. Considering this, we do not explain the present effects in terms of cerebral processing because of the unconventional reference. We have only considered the concordance of results for both devices.

A few studies have already tested these two wireless devices in various contexts, but have reported contradictory results. These studies compared the Epoc + or the g.tech Natilus SAHARA with research-grade devices in a broad range of paradigms such as auditory evoked potential [59], visual decision-making N240 component and motor potential [1]), and visual evoked potential [1]. Comparisons have also been performed in different frequency bands for eyes open vs eyes closed paradigms [60], for a workload task, and for the calculation of signal to noise ratio [60]. Unfortunately, devices have never been tested in musical paradigms. On the one hand, some studies considered that the Epoc + suffers from a large proportion of artefacts [60,61] and that it was under-performant for research applications (e.g investigations into event-related phenomena or time-locked events) but was usable for gaming applications [9,10]. The large proportion of artefacts could be mostly due to the single size of the device, which may not fit every participant’s head [60], and possible oxidation of the platinum, which could also influence the results [10]. On the other hand, others concluded that the both g.tech Nautilus and Epoc + were as good as research-grade devices in many paradigms [1] and demonstrated a sufficient signal quality in terms of ERP peak amplitude and latency [59]. Beyond the signal quality, Radüntz [38], demonstrated that both the g.tech and the Epoc + had interpretable signals in the frequency domain and good signal-to-noise ratio.

For the g.tech device, we do not know of any studies that compare signal quality other than those cited above [1,60]. The device has been used mostly for BCI applications [62,63], for real-time statistical modelling [64], for medical diagnosis [65], in auditory and attention paradigms [66], and so on. Several studies used the Epoc + headset for research in a different context, such as simple emotion detection (Ramirez and Vamvakousis, 2012), detection of emotions elicited by movie clips [67], or for detection of emotional modulation via neurofeedback applications for patients with depression or cancer (Ramirez et al., 2015, 2018). Furthermore, studies by Bevilacqua et al., (2018) and Dikker et al. (2017), suggest that measuring oscillatory cerebral rhythms using the Epoc + can provide reliable data to investigate inter-subject cerebral synchrony.

Although Epoc + has been used and tested for BCI and neurofeedback applications, including for future patient applications [9,10,12–14,48], based on this experiment and other research, we are not sure how these devices can be used for medical purposes. Although it sounds like an interesting solution for routine exams for patients that require a faster positioning for faster EEG exams and better comfort, the g.tech has been reported to be uncomfortable by our participants and those in other studies [6], and the unconventional referencing of Epoc + does not facilitate comparisons with already known cortical patterns associated with specific pathologies. Furthermore, Epoc + is not certified for medical uses.

### Limitations

Firstly, the difficulty of reproducing three listening sessions giving enough chill reports and consequently enough epochs for the analysis is a major concern. This requirement reduced the number of participants who could be included in the analysis. However, it should be noted that the number of reported chills was not different across listening sessions, reducing the possibility of a potential habituation effect. Our participants reported that the g.tech SAHARA pin electrodes were uncomfortable, which is in line with observations elsewhere [6], and this led to unpleasant recording sessions for some participants. Even if some electrodes and regions of interest showed the same effect and trends in both device, a greater sample of subjects and more repetitions of the variable of interest (the chill) might have produced more robust results. The major constraint of wireless devices was the signal quality, which could not be well managed due to the low precision of the impedance measures for both devices. More importantly, the impedance of the g.tech equipped with SAHARA electrodes can be observed but not improved. For the Epoc + headset, good hydration of the saline electrodes on long-term recordings (more than 30 minutes) to give the best signal/noise ratio is still a major concern. Moreover, the effect on central sites for the theta frequency band found in our first study cannot be reproduced considering the absence of electrodes on central sites for the Epoc +. We compared activities from the central ROI from EGI with activities found in Emotiv™ FC5 and FC6 electrodes, but the positioning is relatively different and could be a source of variations. This may explain the difference in the results for these sites.

Further, for the g.tech device, with the adjustable electrode positioning, the electrodes positioned on F7 and F8 should have been positioned on C1 and C2. Consequently, comparison with the EGI central sites was not possible. Additionally, large regions of interest regrouping up to ten electrodes for the EGI compared with isolated electrodes for the wireless devices could be a factor of influence explaining some incongruent results and non-significant trends. Duvinage et al., (2013) also mentioned that the low number of electrodes and their positioning is a major constraint of the Epoc + headset, as is the unconventional referencing, even if—as in our case for emotion detection—the positioning of the frontal and prefrontal electrodes is an advantage.

We observed different results compared to our previous research that used the same data [35] and, despite a smaller sample, the only difference came from the unconventional referencing to P3 P4 for Epoc +. Considering those previous results, the increased significance with Epoc + in the present study does not mean that EGI is less performant, but only that the re-referencing largely shapes the signal. Thus, the reference in P3 and P4 still a major concern for the study of specific cognitive processing with Epoc + devices.

Because of the low number of electrodes and the different positioning of electrodes, it was impossible to use the same re-referencing for all three devices. However, the concordance of g.tech with Epoc + was not an aim of the study and was not performed. This specific paradigm raised the problem of the sequential recordings that potentially induced a bias in measures. By subtracting a baseline period recorded for each listening session, the multiple task related measures biais could have been corrected. In this study, we did not take into account the gender and the age of participants although this can be a factor of variations for EEG and for musical emotions. Further investigations involving both male and female of various age classes would clarify potential differences.

Finally, we did not perform a simultaneous recording with both devices in the same listening session because the Epoc + has a solid montage. The recording of data during two different sessions, even in exactly the same conditions, can be a source of variation [1].

## Conclusion and perspectives

Even if significant congruent effects between devices are low, the concordance of localized activity—especially in the left prefrontal area—and the same localized trends in the alpha frequency band encourage us to validate the Epoc + system for the recording of musical pleasure. For the concordance analysis of g.tech devices, the absence of significant consistent effects with the EGI does not validate the use of this device in such a paradigm. With the development of wireless headsets, fast and easy handling headsets, new perspectives are open for the study of social and affective neurosciences in multi-subject paradigms [68] in natural or ecological musical settings. The study of group dynamics both with subjective responses and objective neurophysiological devices would provide relevant and as-yet-unknown data about cerebral processing associated with behavior and even feeling/emotions [34,69].
